# Molecular cloning, characterisation and mRNA expression of the ryanodine receptor from the peach-potato aphid, *Myzus persicae*

**DOI:** 10.1016/j.gene.2014.11.035

**Published:** 2015-02-10

**Authors:** B.J. Troczka, A.J. Williams, C. Bass, M.S. Williamson, L.M. Field, T.G.E. Davies

**Affiliations:** a Biological Chemistry and Crop Protection Department, Rothamsted Research, Harpenden, Hertfordshire AL5 2JQ, UK; b Institute of Molecular & Experimental Medicine, Cardiff University School of Medicine, Wales Heart Research Institute, Heath Park, Cardiff CF14 4XN, UK

**Keywords:** Ryanodine receptor, Insect, Diamide insecticides, Alternative splicing, Aphid

## Abstract

The peach potato aphid, *Myzus persicae*, is one of the most important agricultural pests of temperate climates. It is mainly controlled through the judicious application of insecticides; however, over time, aphids have developed resistance to many insecticidal classes. The recent introduction of synthetic diamide insecticides, with a novel mode of action, potentially offers new tools to control aphid populations. These diamides act on the ryanodine receptor (RyR), a large endoplasmic calcium release channel. In this study we have cloned cDNAs encoding the complete open reading frame of the RyR from *M. persicae*. The open reading frame is 15,306 base pairs long and encodes a protein of 5101 amino acids. The aphid RyR shares many of the features of other insect and vertebrate RyRs, including a highly conserved transmembrane region. However, unlike the other RyRs characterised to date, the *M. persicae* channel does not display alternative splicing at any stage of its developmental cycle, so it cannot generate functional variants of the channel.

## Highlights

• We obtained the full-length cDNA sequence of the *M. persicae* RyR.
• The ORF is 15,306 bp long and encodes a protein of 5101 amino acids.
• The aphid RyR shares many common features with other insect and vertebrate RyRs.
• The aphid channel does not display alternative splicing at any developmental stage.
• Aphids cannot generate variants of the RyR channel, unlike other insects.

**Abbreviations**
ATP: adenosine triphosphate
BLAST: basic local alignment search tool
bp: base pair(s)
Ca^2 +^: calcium (ions)
CaM: calmodulin
cDNA: DNA complementary to RNA
*E. coli*: *Escherichia coli*
EF hand: the EF hand is a helix–loop–helix structural domain or motif found in a large family of calcium-binding proteins
InsP_3_R: inositol 1,4,5 triphosphate receptor(s)
kb: kilobase(s) or 1000 base pairs
kDa: kiloDalton
MIR: The MIR domain is named after three of the proteins in which it occurs: protein mannosyltransferase, inositol 1,4,5-trisphosphate receptor (IP3R) and ryanodine receptor (RyR)
mRNA: messenger RNA
NCBI: The National Center for Biotechnology Information
NH_4_OAc: ammonium acetate
oligo: oligodeoxyribonucleotide
ORF: open reading frame
para: paralytic
PCR: polymerase chain reaction
pfam: protein family
qRT-PCR: quantitative real time polymerase chain reaction
RIH: RyR and IP3R homology domain
RNA: ribonucleic acid
RyR: ryanodine receptor
SNP: single nucleotide polymorphism
TAE: tris/acetate/EDTA
TM: transmembrane
UV: ultraviolet

**Nucleotide symbol combinations**
Pairs: K = G/T; M = A/C; R = A/G; S = C/G; W = A/T; Y = C/T
Triples: B = C/G/T; D = A/G/T; H = A/C/T; V = A/C/G; N = A/C/G/T

## 1 Introduction

Ryanodine receptors (RyRs) are calcium release channels mainly located on the endo(sarco)plasmic reticulum, with a majority of their mass (consisting of the regulatory domain with multiple binding sites for various ligands) found in the cytosol, whilst the relatively small transmembrane region forms a cation selective ion channel (Samso et al., 2005). Their main role is in regulation of the release of luminal Ca^2 +^ stores into the cytoplasm, hence RyRs are one of the primary components of excitation–contraction (EC) coupling in muscle cells (Fill and Copello, 2002). RyR proteins are homomeric tetramers, with each monomer having a molecular mass of about 500 kDa (Hamilton, 2005). Mammals have 3 isoforms with tissue-specific distribution, whereas insects and other invertebrates have only one isoform. RyRs are closely related to other internal Ca^2 +^ release channels, the inositol 1,4,5 triphosphate receptors (InsP_3_R); however their regulation varies substantially from InsP_3_Rs (Meur et al., 2007).

For decades ryanodine receptors have been considered a potential target for the development of insecticides with a novel mode of action (Nauen, 2006), primarily due to the low overall amino acid identity (approximately 45% homology) between insect and mammalian channels. Initial attempts to develop commercial compounds based on the natural plant alkaloid ryanodine, which acts on RyRs, were unsuccessful due to unacceptable levels of mammalian toxicity (Nauen, 2006; Sattelle et al., 2008). However, the discovery and development of flubendiamide, a synthetic diamide which selectively activates insect RyRs, led to a successful commercialization by Bayer CropScience AG (Ebbinghaus-Kintscher et al., 2007). This was closely followed by the development and commercial release of chlorantraniliprole and cyantraniliprole by DuPont USA, which are also selective RyR activators (Cordova et al., 2006; Lahm et al., 2007). Both flubendiamide and chlorantraniliprole are primarily used to control pests from the order Lepidoptera, whilst cyantraniliprole also shows promise for the control of sucking pests such as aphids and whiteflies (Legocki et al., 2008). These diamides display no cross resistance with any other currently used insecticides, making them excellent tools with which to control insect populations that are resistant to other classes of chemicals (Nauen, 2006). Unfortunately, the first report of field resistance to these compounds was recorded shortly after they were marketed, with a highly resistant strain of diamondback moth, *Plutella xylostella*, being found in China (Wang and Wu, 2012). This was quickly followed by reports of control failures for *P. xylostella* in Thailand and the Philippines (Troczka et al., 2012). Sequencing of the transmembrane region of the RyRs isolated from resistant *P. xylostella* strains collected from these regions identified mutations resulting in an amino acid substitution (G4936E), which appears to confer diamide resistance (Troczka et al., 2012; Guo et al., 2014).

In the present study we report the cloning and characterisation of the cDNA encoding the complete open reading frame (ORF) for the RyR from the peach potato aphid, *Myzus persicae*. This aphid is a major agricultural pest of temperate climates, causing damage to a wide range of economically important crops through direct feeding and the transmission of plant viruses (Blackman and Eastop, 2000). *M. persicae* has developed resistance to many insecticide classes including to the pyrethroids (Devonshire et al., 1998) and neonicotinoids (Bass et al., 2011) which have been the most successful aphid control agents to date. The development of the diamide insecticides offers a new route to control this pest, particularly with the development and registration of cyantraniliprole (Selby et al., 2013).

## 2 Materials and methods

### 2.1 Insects and chemicals

Two clones of *M. persicae*, 4106A — an insecticide susceptible clone of UK origin and 5191A — a neonicotinoid-resistant clone originating from Greece, were reared in Blackman boxes on Chinese cabbage leaves (*Brassica rapa Spp*) at 18 ± 1 °C, 70% relative humidity.

Chemicals were purchased from Sigma unless stated otherwise.

### 2.2 Amplification and sub-cloning of *M. persicae* RyR cDNA fragments

Total RNA was extracted from mixed populations of the two *M. persicae* clones at different developmental stages (including winged/non-winged forms) using an E.Z.N.A. Mollusc RNA kit (Omega Bio-Tek), following the manufacturer's protocol. cDNA templates were synthesised from 4 ***μ***g of total RNA using RevertAid Premium transcriptase (Thermo-Fermentas) or Superscript III (Invitrogen) and an Oligo 16 _d_T primer. PCR primer pairs for amplifying five overlapping cDNA fragments (F1a, F1b, F2, F3 and F4) and related sequencing primers (Table 1) were based on a *M. persicae* RyR sequence published in patent US2011/0086345 A1 (Casper et al., 2010). PCR products for cloning were amplified using *pfu* proofreading polymerase (Promega). PCR fragments used for direct sequencing were amplified using a Long Range taq mix (Thermo-Fermentas). All PCR reactions were analysed on 1% (w/v) TAE agarose gels and visualised using ethidium bromide staining and UV light. PCR products were purified using a QIAGEN gel extraction kit following the manufacturer's guidelines. If the reaction yielded a single product, a 4 M NH_4_OAc/ethanol precipitation was used to recover the fragments.

**Fig. 1:**
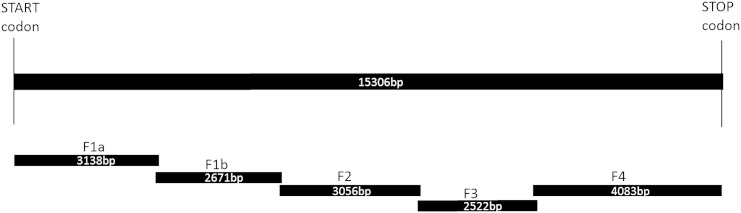
Summary of the strategy adopted to obtain the *M. persicae* RyR cDNA sequence. Five overlapping fragments (F1a/b, F2, F3 and F4) were initially PCR amplified. Fragments F1a, F1b, F2 and F3 were sub-cloned into pJET1.2blunt vector (Thermo-Fermentas) and fragment F4 was sub-cloned into a TOPO®TA vector (Invitrogen) for sequencing.

Five overlapping fragments (F1a/b, F2, F3 and F4) were PCR amplified, sub-cloned into suitable vectors and sequenced. Fragments F1a, F1b, F2 and F3 were sub-cloned into pJET1.2/blunt vector (Thermo-Fermentas), a component of the CloneJET kit, and fragment F4 was sub-cloned using a TOPO XL cloning kit (Invitrogen) following a 5 minute incubation at 72 °C in the presence of ATP and Dreamtaq polymerase to introduce the TA overhangs needed for successful ligation. A summary of the strategy adopted to obtain the *M. persicae* RyR cDNA sequence is shown in Fig. 1. All plasmids were transformed using XL-10 gold *E. coli* ultra-competent cells (Agilent). Plasmids were recovered using a QIAGEN plasmid mini-prep kit following the manufacturer's guidelines. Sequencing was done in-house using a Big Dye Terminator kit v1.1 (Agilent) and a 3100 Genetic Analyser (ABI Prism) or by Eurofins Scientific. Sequence assembly, in situ vector/insert ligations and multiple alignments were carried out using Vector NTI 10.1 advance (Invitrogen) or Geneious v5.5 (Biomatters Ltd., NZL) software.

### 2.3 Quantitative RT-PCR

Quantitative RT-PCR was used to examine the relative levels of expression of the RyR in four different developmental stages of the *M. persicae* clone 4106A; apterous adults, winged adults and nymphal stages (1st/2nd instar and 3rd/4th instar). The primers (Table 2) were designed to amplify fragments of approximately 125 bp.

Total RNA was prepared from each stage using an Isolate RNA mini kit (Bioline, London, UK), following the manufacturer's recommended protocol. 750 ng of the RNA was used for cDNA synthesis using Superscript III (Life Technologies) and random hexamers (Life Technologies, CA, USA), according to the manufacturer's recommended protocol. PCR reactions (15 ***μ***l) contained: 4 μl cDNA (20 ng), 7.5 μl SensiMix SYBR Kit (Bioline, London, UK) and 0.25 μM of each primer. Samples were run on a Rotor-Gene 6000™ (Corbett Research) using temperature cycling conditions of 10 min at 95 °C followed by 40 cycles of: 95 °C for 15 s, 57 °C for 15 s, 72 °C for 20 s. A final melt-curve step was included post-PCR (ramping from 72 °C–95 °C by 1 °C every 5 s) to confirm the absence of any non-specific amplification. The efficiency of PCR for each primer pair was assessed using a serial dilution of 100 ng to 0.01 ng of cDNA. Each qRT-PCR experiment consisted of three independent biological replicates with two technical replicates for each. Data were analysed according to the _ΔΔ_CT method (Pfaffl, 2001), using the geometric mean of two selected housekeeping genes (actin and the ‘*para*’ voltage-gated sodium channel) for normalisation according to the strategy described previously (Vandesompele et al., 2002). In this analysis the reference developmental stage was adult apterous aphids.

### 2.4 Bioinformatics

*M. persicae* and *Acyrthosiphon pisum* (pea aphid) genomes were data mined (BLAST searched) using the GenOuest server (http://tools.genouest.org/tools/myzus/login) and Aphidbase (http://www.aphidbase.com). The intron/exon boundaries were determined using Spidey (http://www.ncbi.nlm.nih.gov/spidey/) and Softberry FGENESH (http://linux1.softberry.com/all.htm) software and by manual sequence analysis using Geneious v5.5 (Biomatters Ltd).

## 3 Results and discussion

### 3.1 Analysis of *M. persicae* RyR

Analysis of the *M. persicae* RyR cDNA and the predicted protein product identified a single *RyR* subunit made up of 5101 amino acids with a molecular mass of 579.981 kDa. The deduced amino acid sequence (accession number KJ933863) has 46.2% homology with human RyR2 (accession number CAA66975.1) and 75.6% homology to the *Drosophila melanogaster* RyR (accession number NP_476991). The predicted protein also has considerable (> 75%) homology (Table 3) to, and shares common features with, other characterised invertebrate RyRs: a Pfam database search indicates the presence of domains such as MIR (amino acids 212–293), SPRY (663–801, 1091–1214, 1539–1682) and RIH (440–648, 2219–2450), although the function of these remains unknown. A potential EF-hand Ca^2 +^ binding domain pair (4198–4248) was also identified — this domain is also partly conserved in the recently characterised *P. xylostella* RyR (X. Wang et al., 2012). Transmembrane (TM) helix prediction using a hidden Markov approach (Krogh et al., 2001) indicates the presence of 6 TM helices close to the —COOH terminus of the sequence with the probable pore-forming domain being located between TM5 (4897–4919) and TM6 (4977–4996). The sequence motif GXRXGGGXGD, critical for RyR ion conductance, is also fully conserved in the *M. persicae RyR* (Zhao et al., 1999). The amino acid residue Q4863 found in mouse RyR2, and thought to be responsible for sensitivity of the channel to ryanodine, is also conserved in the aphid RyR (Wang et al., 2003). The glycine residue G4946, where a mutation has been linked to a high level of resistance to diamides in *P. xylostella*, is also present in the *M. persicae* RyR (Troczka et al., 2012). Overall the entire transmembrane region is highly conserved.

### 3.2 Aphid RyR gene structure

**Fig. 2:**
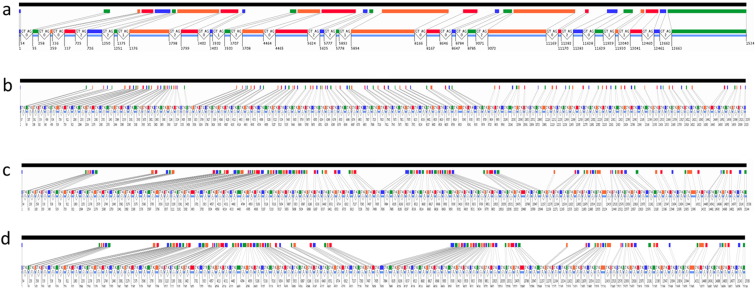
Exon predictions for RyR genes from (a) *Drosophila melanogaster*, (b) *Bombyx mori*, (c) *Acyrthosiphon pisum* and (d) *Myzus persicae*. Images were generated using the MGalign online tool (http://proline.bic.nus.edu.sg/mgalign/mgalignit.html) which generates graphical representations of mRNA to genome alignments. The upper (black) bar represents the genomic sequence. Directly below is a representation of the mRNA sequence and each coloured bar indicates an exon. The size and position of the exon are shown relative to the size of the genomic segment. Below this the coloured bars represent the size and position of the exon relative to the size of the mRNA sequence. The first and last two nucleotides of the introns which constitute part of the splice site motif is shown as is the phase of the intron on the basis of the open reading frame (ORF). Numbers indicate exon start and end positions. The light blue bar below the mRNA sequence represents the ORF.

We used data-mining of the *M. persicae* and *A. pisum* (pea aphid) genome databases to identify scaffolds containing RyR sequence and to identify the intron–exon boundaries. This showed that the RyR genes in both species are structurally similar and consist of 98 exons corresponding to the full ORF sequence of 15,306 bps (Fig. 2). All of the *M. persicae* RyR exons are on a single scaffold occupying a region of 59,441 bps, making the gene significantly larger than the RyR gene of the model insect *D. melanogaster*, that comprises only 26 exons and covers a region of 25,680 bps (Takeshima et al., 1994). Analysis of other RyR genes from other insects with available genome sequences showed that they have a diverse number of exons, with 109 in *Bombyx mori* and 53 in *Apis mellifera* (TGE Davies, unpublished), making some of the insect RyRs comparable to their human counterparts which contain over 100 exons (George et al., 2007). The two aphid RyRs are highly conserved, with 95% similarity at the nucleotide level and 99.3% similarity at the amino acid level.

### 3.3 Sequence polymorphisms

Sequence comparison of the *M. persicae* RyR cDNA with the previously published sequence in patent US2011/0086345 A1 (Casper et al., 2010) identified 44 bps that differed between our coding sequence and the previously published one, with 28 bps resulting in an amino acid change (Table 4) (Casper et al., 2010). Multiple alignments of our predicted protein sequence with *M. persicae* and *A. pisum* genomic sequence annotations and 43 other invertebrate and vertebrate RyR protein sequences suggest that the amino acid variations noted were most likely due to errors in the previously published sequence (Casper et al., 2010). There were 8 silent single nucleotide polymorphisms (SNPs) identified between the predicted (clone G006) genomic sequence and isolated (clones 4106A and 5191A) *M. persicae* RyR cDNA sequences, indicating that the origin of the sequence (different clones from different geographical locations) does not have a significant impact on gene variability. Direct sequencing of the entire *M. persicae* RyR cDNA obtained from mixed developmental stage populations of clones 4106A and 5191A did not detect any additional SNPs. This homogeneity is in direct contrast to the situation in other characterised insect RyRs. For *P. xylostella* six different RyR isoforms have been identified (accession numbers: JN801028.1; JF926694.1; JF927788.1; JX467684.1; JF926693.1; JQ769303.1) with multiple SNPs (Sun et al., 2012; X. Wang et al., 2012). Sequence polymorphisms (both silent and amino acid changing) have also been reported for *Cnaphalocrocis medinalis* (J. Wang et al., 2012).

### 3.4 Splicing of aphid RyR genes

During sub-cloning and sequencing of multiple *M. persicae* RyR cDNA fragments no alternative splice sites were detected, despite the fact that the total RNA used was obtained from a mixed aphid population comprising various developmental stages and two distinct clones (4106A and 5191A). This absence of isoforms is in stark contrast to other characterised insect RyRs, in which a number of splice sites have been identified. Alternative splicing of insect RyRs was first reported in *D. melanogaster*, with 2 different splice sites identified generating alternative mRNA forms (Takeshima et al., 1994). A third splice site was reported on isolation of cDNA for heterologous expression of *D. melanogaster* RyR (Xu et al., 2000). Alternatively spliced RyR genes have subsequently been reported in insects belonging to several different orders including Lepidoptera (*Heliothis virescens* (Puente et al., 2000), *P. xylostella* (X. Wang et al., 2012), *C. medinalis* (J. Wang et al., 2012), *Helicoverpa armigera* (Wang, Liu et al., 2013), *Ostrinia furnacalis* (Cui et al., 2013)), Diptera (*Bactrocera dorsalis* (Yuan et al., 2014)) and Hemiptera (*Nilaparvata lugens* (Wang, Xie et al., 2013)). Some of this splicing results in either sequence deletions or insertions, and appears to be species specific, although one common splice site for two mutually exclusive exons, located within the second SPRY domain (amino acids 1135–1167 of the *M. persicae* RyR), has been identified in many insects (described in detail by Wang et al. (Wang, Xie et al., 2013)). However, we did not detect this splice site in *M. persicae*, and analysis of the corresponding genomic sequence showed that the introns flanking exon 24 are not sufficiently long (with predicted sizes of 63 and 71 bps) to contain the alternative form of that exon. We therefore conclude that the *M. persicae* RyR lacks this common feature present in other insects.

**Fig. 3:**
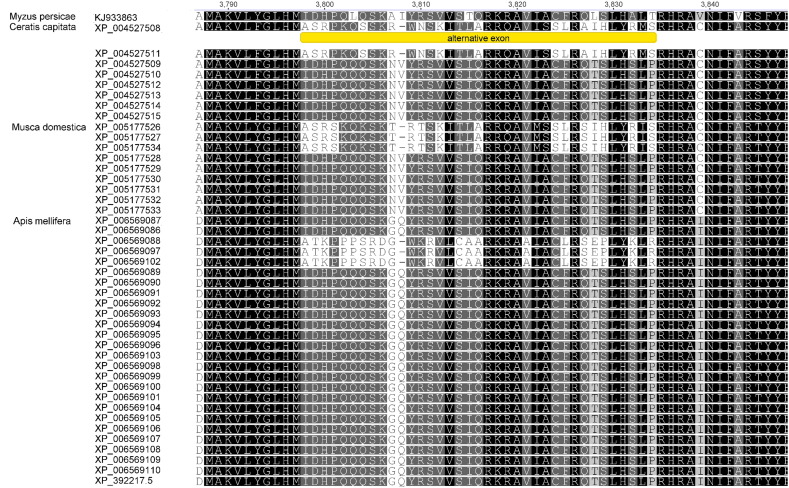
Multiple alignments of computationally predicted RyR isoforms (NCBI database) for *Ceratis capitata* (sequences 2–9), *Musca domestica* (sequences 10–19), and *Apis mellifera* (sequences 20–44), showing a second splice site for two mutually exclusive alternative exons.

A BLAST search of the full NCBI nr database identified further possible RyR splice variants in insects, including the prediction of 26 hypothetical isoforms of the *A. mellifera* RyR protein, 9 for *Musca domestica*, 8 for *Ceratis capitata* and 10 for *D. melanogaster*. Multiple alignments of all of these identified the common splice site in the second SPRY domain as well as a predicted second splice site for two mutually exclusive exons at amino acid positions 3720–3756 of the *M. persicae* RyR, which corresponds to exon 71 in the *M. persicae* gene (Fig. 3). The second splice site would encompass one of the predicted calmodulin (CaM) binding sites in mammalian isoforms (Lau et al., 2014), which is partly conserved in insect RyRs, and the predicted alternative exon would modify the binding site. Since CaM is a known modulator of RyRs, this alternative exon may play a role in regulating channel sensitivity to CaM. Although both flanking introns of exon 71 in *M. persicae* RyR are large enough (3760 and 1499 bps) to contain an alternative exon we were not able to detect it in any of our sequencing or within the intronic sequences.

In humans alternative splicing of the RyR2 isoform is responsible for profound differences in calcium release and overall cell susceptibility to undergo apoptosis and for differentiation of intracellular RyR2 targeting (George et al., 2007). However, to date, the functional implications of alternative splicing in insect RyRs has not been studied in detail. To this end it is very interesting that the *M. persicae* RyR lacks the diversity seen in other insects and vertebrate RyRs.

### 3.5 Expression of the *M. persicae* RyR in different developmental stages

**Fig. 4:**
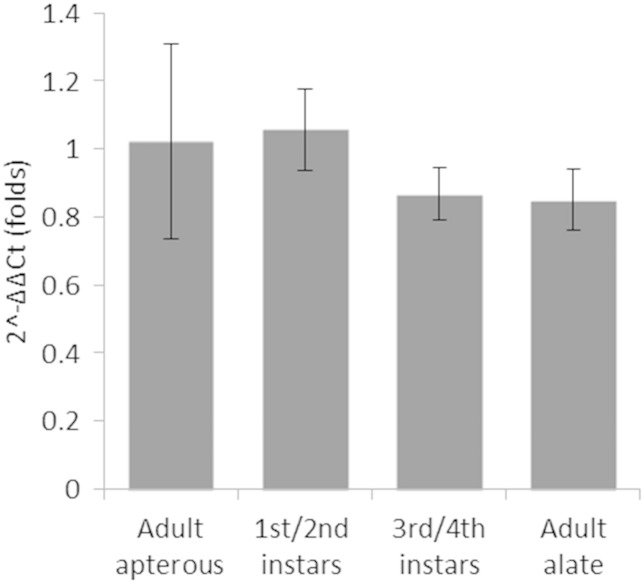
Relative expression (fold change) of the RyR gene at different developmental stages of *M. persicae* clone 4106A. The apterous adult was used as a reference. There were no apparent differences observed between any of the developmental stages tested. The data are presented as a mean with 95% confidence intervals.

The level of expression of the *M. persicae RyR* gene was measured in different developmental stages of clone 4106A, including a mixed pool of first and second instars, mixed third and fourth instars and apterous and winged adults, but no statistically significant differences were found (Fig. 4). This contrasts with other insect species which have differential expression of RyRs during development. For example, in *P. xylostella* expression is significantly different between egg, larval, pupal and adult stages, and also in various body parts of the adult moth, being higher in the thorax and lower in the abdomen (X. Wang et al., 2012). Similar results were found in *H. armigera* with the highest expression in adults and adult heads (Wang, Liu et al., 2013). Differences were also found in expression of RyRs in isolated tissues of fourth instar larvae in another *P. xylostella* study (Guo et al., 2012). Differential expression was also found in *N. lugens* between macropterous female adults and other developmental stages (Wang, Xie et al., 2013). The expression level of different splice forms is also varied between different developmental stages and anatomical body parts in *C. medinalis* (J. Wang et al., 2012). For *M. persicae* a more refined tissue specific analysis of RyR expression levels would be difficult to conduct due to the aphids morphology and the presence of multiple generations within the clonal females.

## 4 Conclusions

We have obtained the complete cDNA encoding the ORF of *M. persicae* RyR and analysed the predicted protein and gene structure. We report a surprising lack of splice sites or SNPs. It remains unclear why the fairly extensive RyR diversity found in other insects is not present in *M. persicae*, but it is possibly the result of its asexual reproductive model. Understanding the RyR of aphids is likely to become more important as they are a target for the diamide insecticide cyantraniliprole. That *M. persicae* is currently susceptible to diamides and there is no cross resistance with any other classes of insecticides was demonstrated in a recent study (Foster et al., 2012). With the risk of resistance emerging in the future a detailed characterisation of the diamide target, as described here, will facilitate the rapid characterisation of future resistance and is a first step towards the development of insecticide screening tools based on heterologous expression of the RyR.

## Figures and Tables

**Table 1:** Primer pairs used for amplification of individual PCR fragments.

PCR product	Primer name	Sequence 5′-3′
F1a	F1a-F	ATGGCCGACAGCGAGGGCAGTTCG
	F1a-R	GTGCGGACTGTTTCGCTTGCTGTATCTC
F1b	F1b-F	GCTAATAGAGATACAGCAAGCGAAAC
	F1b-R	ACAGCCATGGTCGAGAAACAGTTGC
F2	F2-F	CTTGAGGAAGCCGTATTAGTTAACCAGG
	F2-R	CCTCTATTGTTCAAAGGTGCATCGTTC
F3	F3-F	GGATGGAGTATTGAACATTCAGAGAACG
	F3-R	GCGTGGAGTGAGAGTTGTCGGAAACA
F4	F4-F	CGAGGTTGCGGATAGAATTGTTGCT
	F4-R	TCAGACGCCTCCTCCGCCGCCGAGCT

**Table 2:** qPCR primer pairs used to determine the level of expression of RyR in different developmental stages of *M. persicae*.

Primer	Sequence 5′-3′	Expected amplicon size
Mz.qPCR3-F	CGAACTTGCATTAGCGTTGA	125 bp
Mz.qPCR3-R	CTGGATCCCAGCCTAAATCA	
Mz.qPCR4-F	CAATTGGGAATCGCAGTTCT	127 bp
Mz.qPCR4-R	CGCTGCACGAGTTCATTAAA	

**Table 3:** Comparison of insect RyR protein sequences (shown as % identity).

	*D. melanogaster*	*P. xylostella*^a^	*B. mori*^b^	*A. pisum*^b^	*A. gambiae*	*B. tabaci*	*C. medinalis*	*S. exigua*	*A. mellifera*^b^	*M. persicae*
*D. melanogaster*	x	77.8	78.9	75.6	82.4	77.1	79.3	78.9	78.8	75.6
*P. xylostella*^a^		x	91.2	77.0	78.5	78.7	92.0	92.1	80.7	77.1
*B. mori*^b^			x	77.7	79.6	79.3	93.8	94.6	81.7	77.8
*A. pisum*^b^				x	76.9	82.3	78.3	78.3	79.7	99.3
*A. gambiae*					x	77.9	80.2	80.0	79.4	77.0
*B. tabaci*						x	79.9	79.9	82.4	82.3
*C. medinalis*							x	95.0	82.3	78.2
*S. exigua*								x	82.3	78.4
*A. mellifera*^b^									x	79.6
*M. persicae*										x

a First published sequence.

b Automated computational prediction by NCBI.

**Table 4:** SNPs found in the *M. persicae* RyR sequence published in patent US2011/0086345 A1 (Casper et al., 2010). Out of 44 polymorphisms 28 result in an amino acid change.

DNA position	Patent	Experimental	AA change	Protein position
453	C	A	N–K	151
479	C	G	P–R	160
675	C	T		
1892	A	C	Q–P	631
2137	A	G	N–D	713
2195	G	A	G–E	732
2454	C	T		
2549	G	A	G–D	850
2712	G	A		
2972	G	A	G–E	991
3696	C	T		
3721	G	A	A–T	1241
3756	G	A		
3923	G	A	G–D	1308
3943	G	A	G–R	1315
4019	G	A	R–Q	1340
4414	C	T	P–S	1472
4771	G	A	G–R	1591
4854	A	G		
5531	N	A	X–N	1844
5548	T	C		
6287	G	A	R–K	2096
6817	G	A	V–M	2273
6830	T	C	P–L	2278
7577	A	G	Y–C	2526
7842	G	A		
7938	G	T	K–N	2646
7988	G	A	G–D	2663
9166	C	T	H–Y	3056
9733	G	C	D–H	3245
10031	C	T	T–M	3344
10592	G	A	S–N	3531
10973	G	A	R–Q	3658
11004	C	T		
11347	G	A	V–M	3783
11394	A	G		
12221	T	A	L–Q	4074
12546	T	A		
12819	G	A		
13227	T	C		
13398	T	C		
14241	A	G		
14414	C	T	S–L	4805
15033	G	A		
